# Dr. Thomas Dwight

**Published:** 1911-11

**Authors:** 


Dr. Thomas Dwight, the successor of Dr. Oliver Wendell Holmes, in the Parkman professorship of anatomy at Harvard, died September 8, 1911. While Dr. Holmes was noted for his ready wit—though it was always of a kindly and instructive nature—Dr. Dwight was a strict disciplinarian and never permitted levity in his classes.

